# Supplementary material for: Lineage-specific differences and regulatory networks governing human chondrocyte development
Source: eLife. 2023 Mar 15;12:e79925. doi: 10.7554/eLife.79925 (PMC10069868; doi:10.7554/eLife.79925)
Supplement: Supplementary file 6. [file elife-79925-supp6.docx]

Supplementary File 6. Oligonucleotides

| REAGENT or RESOURCE | SOURCE | IDENTIFIER |
| --- | --- | --- |
| qPCR primers for RNA expression | | |
| TBP : Forward : TGAGTTGCTCATACCGTGCTGCTA  Reverse : CCCTCAAACCAACTTGTCAACAGC | This paper | N/A |
| PRG4: Forward : ACCACCACCAGACCTAACCAAACT  Reverse : TGAGGTGTTTCTCCTTCAGCACCA | This paper |  |
| COL10A1 : Forward : AAAGGCTACCTGGATCAGGCTTCA  Reverse : ATAGGCCATTTGACTCGGCATTGG | This paper |  |
| PTH1R : Forward : GAGTGGGAGACAGTCATGTG  Reverse : GGAAATCATTCAACCACCCATC | This paper |  |
| PTHLH : Forward : CAAGGTGGAGACGTACAAAGAG  Reverse : CCCAGTCACTCCAGAGTCTAA | This paper |  |
| FGF18 : Forward : CGTGACCTAGTACACCAATGATAA  Reverse: ACTGCAATATACAGAGAGGTGAAA | This paper |  |
| FGFR3 : Forward : GAAGGTTTATCCCGCCGATAG  Reverse: CACTGGAATCACCTCCAACTATTA | This paper |  |
| RELA : Forward : TGAGCCCACAAAGCCTTATC  Reverse : ACAATGCCAGTGCCATACA | This paper |  |
| RUNX2 : Forward : AGTTTGTTCTCTGACCGCCTCAGT  Reverse : AAAGGACTTGGTGCAGAGTTCAGG | This paper |  |
| MEOX1 : Forward : GTCAACGTGAGTTTGGATCTCT  Reverse : CCACAGCTCTTGCCCTTT | This paper |  |
| CHI3L1 : Forward : CTCTATCACCAAGGAGCCAAA  Reverse : CGTTCCTAAGTGAAGGTTTCAAG | This paper |  |
| PANX3 : Forward : CGTTTATGGAGGCAATTCCATATC  Reverse : GATTGCCTCACTTGCTCTCT | This paper |  |
| ALPL : Forward : CCTTGCTCACTCACTCACTC  Reverse : CTGCCAGGTGGCTCTTC | This paper |  |
| GLIPR2 : Forward : GGAAGGTGGCAGACTTAAGAA  Reverse : CATCACACACACAAGCACATAC | This paper |  |
| LOXL2 : Forward : CCAACGTGGCCAAGATTCA  Reverse : CCACTGGCCATCATAGTACAC | This paper |  |
| DKK3 : Forward : GCTGTGGGTAGATGTGCAATA  Reverse : CTGGGAAGAAGATGTAGGAAGAAG | This paper |  |
| TLR2 : Forward : CACTGGACAATGCCACATATC  Reverse : CACAAGACAGAGAAGCCTGATT | This paper |  |
| LTBP2 : Forward : GGTTACTGCGAGAACACAGA  Reverse : GAGGCCATTTCCAGGTAGTT | This paper |  |
| COL15A1 : Forward : TGAACGCATCCCAACATAGG  Reverse : AGGACATCTTACACTCAACAACA | This paper |  |
| ATOH8 : Forward : CCTGAGACTATCCCAGAAGAGA  Reverse : TGGAGACAGGAAGGAGCATA | This paper |  |
| ACAN : Forward : CCAAGAGCAGTGCAATCGTTGGTT  Reverse : ATACATTCAGCTGCGGTTCCGAGT | This paper |  |
| C16orf72 : Forward : AAGTTCCTCCACCACGAAAC  Reverse : AGGGTTGCAAATCAGTCTCTAC | This paper |  |
| RCL1 : Forward : CCTGTGTTGGCATTGGTTTC  Reverse : AGCTTCTGCTTTGTCTGTAGG | This paper |  |
| WNT10B : Forward : AGAGTGGGTGAATGTGTGTAAG  Reverse : GAGTGACCTTGGAAGGAAATCA | This paper |  |
| GPR153 : Forward : GCTTCATCGTGGCTGAGAT  Reverse : CGATGGCTGTGCAGATCA | This paper |  |
| MAP4K3 : Forward : AGCTGCAGTAACCTGTCTTC  Reverse : AAGCACTTGTGTGGTTCAATATG | This paper |  |
| RXRA : Forward : GCCTGGAACATCTCTTCTTCTT  Reverse : CCGCAGGCCTAAGTCATTT | This paper |  |
| SCUBE1 : Forward : GGACGAGTGTCAGGACAATAA  Reverse : GCTGGTTGTCACTAAGGAAGA | This paper |  |
|  |  |  |
|  |  |  |
| **ChIP-qPCR Primers (from ActiveMotif)** | | |
| hDPF1 peak positive control for RUNX2 binding :  Forward : GCTGTGCCAACCACTTTCTAC  Reverse : CCGTTCTGCTGTGGGTAATG | Active Motif | h DPF1_+16kA,  h DPF1_+16kB |
| hBIRC3 peak positive control for RELA binding :  Forward : GCTTTTGGGTCATGGAAATC  Reverse : TCCCCACCCCTATCTGTACC | Active Motif | h BIRC3-24 A,  h BIRC3-24 B |
| Putative COL15A1 peak bound by RELA :  Forward : GGCTACTTGGTGCTTCAGGA  Reverse : CCCTCAATGGTCGCTTTTTA | Active Motif | h COL15-804 _A,  h COL15-804 _B |
| Putative PRG4 peak bound by RELA :  Forward : CTTGCCTTACTCCTGCCTTTAG  Reverse : TGGATATGGATCTGCTGTTTG | Active Motif | h_PRG4-64K_A,  h_PRG4-64K_B |
| Putative LTBP2 peak bound by RELA :  Forward : AAAACCCGGCAGAGAGAGTC  Reverse : TGTGTCTGGGCAAACCTTTC | Active Motif | h LTBP2 -4.5K_A,  h LTBP2 -4.5K_B |
| Putative GLIPR2 peak bound by RELA :  Forward : CTGTCGGGATGAACTTTCTG  Reverse : GGACTGGAGTGTGTCCTTTC | Active Motif | h_GLIPR2+574_A,  h_GLIPR2+574_B |
| Putative DKK3 peak bound by RELA :  Forward : TGTTCTCAAGCAGCAGACATG  Reverse : CCTCTTGGGGAAACTCACAG | Active Motif | h_DKK3-71K_A,  h_DKK3-71K_A |
| Putative TLR2 peak bound by RELA :  Forward : TTCATGTCCCGCAATGTAGAG  Reverse : CTTGACCCCACCTTGTTTCTC | Active Motif | h_TLR2+78K_A,  h_TLR2+78K_B |
| Putative LOXL2 peak bound by RELA :  Forward : CGCAGCACTTGGTTTCTCTC  Reverse : GCCTCGTTGCCAGTACAGTG | Active Motif | h_LOXL2+43K_A,  h_LOXL2+43K_B |
| Putative ACAN peak bound by RUNX2 :  Forward : TCCCAACCAAACCTCTCTTG  Reverse : TTCACCCAGGCCAGTAAGAC | Active Motif | h_ACAN-35K_A,  h_ACAN-35K_B |
| Putative ATOH8 peak bound by RUNX2 :  Forward : TTTGACGACCCGTTCCTATG  Reverse : CGGAGAACAACGCCTAAGTC | Active Motif | h_ATOH8-692_ A,  h_ATOH8-692_ B |
| Putative C16orf72 peak bound by RUNX2 :  Forward : TTTACATGCCGTGGTGAATC  Reverse : GGGCCAGCGAGAAAGTTAAG | Active Motif | h_Cl6ORF72-18K_A,  h_Cl6ORF72-18K_B |
| Putative COL10A1 peak bound by RUNX2 :  Forward : CCTCCCAAAGCTACCCTGTC  Reverse : CAACCAAAATCTAACCTGTGTTC | Active Motif | h_COL10A1-3702_A,  h_COL10A1-3702_B |
| Putative RCL1 peak bound by RUNX2 :  Forward : CCCCAGGAGAGTTGACACAG  Reverse : AGCAGTGGCTGAAATGTTTG | Active Motif | h_RCL145k_A,  h_RCL145k_B |
| Putative WNT10B peak bound by RUNX2 :  Forward : GTGTCATCAAGGGCAAGTA  Reverse : CAGTGTGAGTGGGGAACTC | Active Motif | h_WNT10B+802_A,  h_WNT10B+802_B |
| Putative GPR153 peak bound by RUNX2 :  Forward : GAGCAGCGTGAATCCGTAAC  Reverse : GCTTCTAGGAACCGGGAATC | Active Motif | h_GPR153-578_A,  h_GPR153-578_B |
| Putative MAP4K3 peak bound by RUNX2 :  Forward : AAGGAGGTGGTGGCTAGTTTC  Reverse : TCACTGGGTTTATGCGTCAG | Active Motif | h_MAP4K3-54K_A,  h_MAP4K3-54K_B |
| Putative RXRA peak bound by RUNX2 :  Forward : GCAAATGTGGATGCTTGTTG  Reverse : GTACTGCTCCTTGGGGACAG | Active Motif | h_RXRA-279K_A,  h_RXRA-279K_B |
| Putative SCUBE1 peak bound by RUNX2 :  Forward : GCCCACTTCCCACAAATAAAG  Reverse : CACCTCATCCCAACCACATAC | Active Motif | h_SCUBE1-37K_A,  h_SCUBE1-37K_B |
| Untr12: Human negative control primer set 1 | Active Motif | Catalog#71001 |
